# The Complete Chromosome‐Level Genome and Mitogenome Assembly of *Seriola lalandi lalandi* (Yellowtail Kingfish) for Aquaculture and Fisheries Management

**DOI:** 10.1002/ece3.74430

**Published:** 2026-10-11

**Authors:** Carla H. Finn, Vinko Besic, Tom Oosting, Yvan Papa, Alvin Setiawan, Maren Wellenreuther, David Chagné, Peter Ritchie

**Affiliations:** ^1^ Te Herenga Waka—Victoria University of Wellington Wellington New Zealand (Aotearoa); ^2^ New Zealand Institute for Public Health and Forensic Science Porirua New Zealand (Aotearoa); ^3^ Earth Sciences New Zealand Ruakākā New Zealand (Aotearoa); ^4^ Bioeconomy Science Institute Lincoln New Zealand (Aotearoa); ^5^ School of Biological Sciences The University of Auckland Auckland New Zealand (Aotearoa)

**Keywords:** aquaculture, assembly, fisheries, genome, *Seriola lalandi*

## Abstract

*Seriola lalandi* comprises three genetically distinct clades, with *S. lalandi lalandi* inhabiting the Southern Hemisphere. While a chromosome‐level genome exists for the Northwestern Pacific *S. lalandi aureovittata*, no such resource is available for *S. lalandi lalandi* , limiting genetic research and aquaculture advancements. This study presents a high‐quality chromosome‐level genome assembly of *S. lalandi lalandi* using Illumina short‐read, long‐read (ONT) and Hi‐C. A total of 289.12 Gb of raw genomic data were generated, and a final assembly of 644 Mb was produced with 99.64% of the assembled bases anchored into 24 pseudo‐chromosomes. Genome annotation identified 24,600 protein‐coding genes, with 96.1% completeness based on 3640 Benchmarking Universal Single‐Copy Orthologues (BUSCO). Additionally, the complete mitogenome was assembled and annotated for *S. lalandi lalandi* . This genomic resource will help to inform comparative genomic studies, improve aquaculture breeding programmes, and support appropriate fishery strategies, particularly in Aotearoa New Zealand, where *S. lalandi lalandi* is a culturally significant species for Māori.

## Graphical abstract

This study reports the first chromosome‐level genome assembly for the Southern Hemisphere subspecies Seriola lalandi lalandi, addressing a critical gap in genomic resources previously limited to the Northwestern Pacific subspecies. The 644 Mb assembly, generated using integrated Illumina, Oxford Nanopore and Hi‐C sequencing, demonstrates high completeness and contiguity, providing a robust genomic foundation to support comparative genomics, aquaculture improvement and fisheries management, particularly in Aotearoa New Zealand where the species is of cultural significance to Māori.

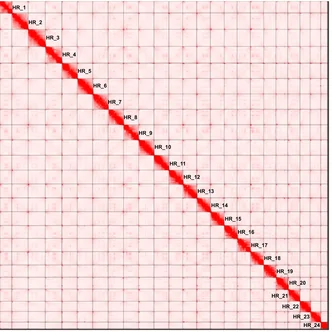

## 1 Introduction

*Seriola lalandi* (Figure 1) is a circumglobally distributed marine pelagic piscivore. Genetic analyses have identified three distinct clades, each corresponding to a geographic region: the Northwestern Pacific (*S. lalandi aureovittata*), the Northeastern Pacific (*S. lalandi dorsalis*) and the Southern Hemisphere ( *S. lalandi lalandi* ) (Premachandra et al. 2017). This population structure is not only relevant to understand the evolutionary history of the species, but also has practical implications for aquaculture, since farmed stocks are typically derived from wild populations specific to the region. Collectively, these clades form an important component of global aquaculture production, owing to their rapid growth and high market value (Symonds et al. 2012).

**FIGURE 1:**
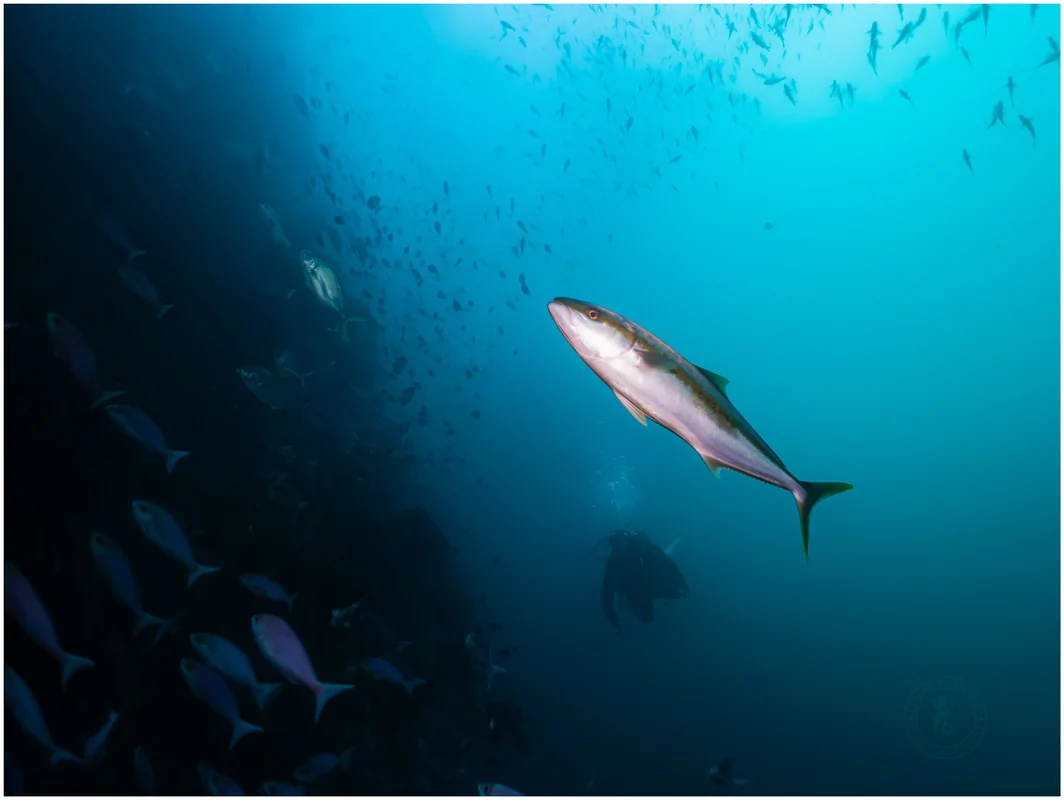
A Yellowtail kingfish from Northland, Aotearoa New Zealand. *Source:* Photograph by Lukas Phan‐huy, via iNaturalist NZ, licenced under CC BY‐NC 4.0. Observation date: 28 September 2024, Northern Arch, Poor Knights Islands, New Zealand.

In aquaculture, high‐quality reference genomes are a critical resource for selective breeding and genetic improvement programmes. They enable the discovery of genetic markers associated with economically important traits such as growth rate, feed efficiency, flesh quality and disease resistance (Houston et al. 2020; Yáñez et al. 2023). While a chromosome‐level reference genome assembly exists for the Northwestern Pacific *S. lalandi aureovittata* population (Li et al. 2022), no equivalent assembly is available for Southern Hemisphere *S. lalandi lalandi* . This gap is significant because the use of a reference genome from a genetically divergent population can introduce reference bias—systematic errors in read mapping and variant calling that arise when sample genomes differ substantially from the reference sequence (Günther and Nettelblad 2019). Such biases can obscure true genetic variation and reduce the accuracy of downstream analyses. Recent studies underscore the value of contemporary and population‐specific reference genome assembly in reducing reference bias, provided genome quality is not compromised (Thorburn et al. 2023).

Given these considerations, a high‐quality de novo reference genome assembly using a contemporary *S. lalandi lalandi* individual is preferable for accurate and confident studies of the Southern Hemisphere population. Such an assembly will capture the unique genetic characteristics, diversity and adaptations of the local population, providing a critical foundation for selective breeding, disease resistance research and stock management in aquaculture sourcing the local population. While this is particularly valuable for advancing sustainable aquaculture practices in the Southern Hemisphere, it also has broader conservation implications. Notably, as climate change drives rapid distributional shifts, hybridization across *Seriola* species is becoming more common (Takahashi et al. 2021), further underscoring the need for population‐specific genomic resources to investigate and monitor population‐level changes.

### 1.1 Cultural Acknowledgement and Context

#### 1.1.1 Naming of the Genome—Haku Raukura

The individual used to generate this genome was sourced from Te Akau (Bream Bay—Ruakākā, Aotearoa New Zealand), which is within the traditional territorial region (‘rohe’) of Patuharakeke Te Iwi Trust hapū (kinship group). Under New Zealand's Treaty framework and established Indigenous data governance principles, Patuharakeke retain custodial authority (‘kaitiaki’) over biological resources originating from their region and over genomic data derived from those resources. We acknowledge the guidance of Te Pou Taiao o Patuharakeke Te Iwi Trust throughout the research process, and are grateful that the final *S. lalandi lalandi* genome has been named Haku Raukura by Patuharakeke Pou Ahurea Roopu, a collective of kaumatua (elders) and tohunga (experts). The name reflects a narrative of chiefly strength and spiritual significance: haku, the Māori name for *S. lalandi lalandi* , and raukura refers to a feather, plume or treasure, traditionally worn by individuals of high rank. The raukura, as a symbol of mana (power/status) and prestige, is likened to the haku—an ika (fish) renowned for its strength, agility and fighting spirit. The name Haku Raukura thus embodies a fusion of physical prowess and cultural reverence, affirming the genome's status as a taonga tuku iho (treasured inheritance) and symbolises the indelible link between haku and tangata (people).

#### 1.1.2 Data Availability and Sovereignty

The *S. lalandi lalandi* individual used in this study was sourced from within the rohe (region) of Patuharakeke iwi, hapū and whanau (tribes, kinship groups and families). Therefore, Patuharakeke rightfully holds responsibilities of kaitiakitanga (guardianship) over their rohe and, by extension, over the species and individuals that originate from it—including the individual used in this study, and including the raw and analysed data derived from it. Patuharakeke's authority over their *S. lalandi lalandi* and resulting *Haku Raukura* (genome assembly) gives effect to the Treaty of Waitangi, a treaty signed in 1840 between Māori chiefs and the British Crown. Patuharakeke Te Iwi Trust have expressed that the use of such taonga (treasure) must align with the principles of Wai 262/Ko Aotearoa Tēnei, which affirms the rights of Māori to protect and control their cultural heritage and biological resources. Therefore, the data is made available *by request* on the Aotearoa Genomics Data Repository (AGDR) (Te Aika et al. 2023), with permissions granted by the Patuharakeke Te Iwi Trust. The AGDR has been developed to follow the principles of Māori Data Sovereignty, and to enable kaitiakitanga (guardianship), so that can effectively exercise their responsibilities as guardians over biological entities that are taonga (treasured). Internationally, Māori ownership and control of natural resources are supported by the UN Declaration on the Rights of Indigenous Peoples 2007.

## 2 Materials and Methods

### 2.1 Sampling and Tissue Extraction

Whole blood and liver tissue was collected from a captive‐bred *S. lalandi lalandi* (haku/yellowtail kingfish) individual (likely F1 or F2 generation from wild‐caught broodstock; sourced by NIWA from Te Akau/Bream Bay, Ruakākā, Aotearoa New Zealand) (885 g, 38 cm, 344 days old, female). Tissue samples were obtained 0.5–1.5 h post mortem following anaesthetic overdose. High‐molecular‐weight DNA was extracted from whole blood using a high‐salt extraction protocol adapted from Aljanabi and Martinez (1997) and Oosting et al. (2020), employing the Nanobind CBB Big DNA Kit (Circulomics). Prior to long‐read sequencing, the integrity of DNA fragments was assessed by gel electrophoresis in 1% agarose. DNA quantity was also assessed using the Quant‐iT PicoGreen dsDNA assay kit, while purity and quality were assessed using the NanoDrop (ThermoFisher) and a Genomic DNA ScreenTape (Agilent), respectively. DNA samples had concentrations > 200 ng/μL, *A*_260/280_≈1.8, *A*_260/230_≈2 and total yields > 20 μg.

### 2.2 Sequencing

Long‐read sequencing was performed at PHF Science (the New Zealand Institute for Public Health and Forensic Science) using Oxford Nanopore (ONT) sequencing on the MinION (SQK‐LSK112) and PromethION (SQK‐LSK114) instruments with R10 chemistry, generating 94.18 Gb of raw ONT sequence data. Additionally, genomic DNA was also sent to AGRF (Australian Genome Research Facility, Melbourne, Australia) for Illumina paired‐end sequencing on the NovaSeq, with 150 bp read lengths, generating 79.42 Gb of raw Illumina data. Liver tissue was sent to BGI Tech Solutions Co. Ltd. (Hong Kong, China) for genomic DNA extraction, Hi‐C library preparation, and sequencing on the DNBseq platform with paired‐end (PE) reads of 2 × 150 bp. This resulted in 115.32 Gb of data with a Q20 (%) of 97.8.

### 2.3 Quality, Contamination and Mitochondrial Filtering

#### 2.3.1 ONT Reads

DNA was sequenced using both the PromethION and MinION platforms, and base‐calling was performed using different algorithms based on ONT's software compatibility: The MinION sequencing data were base‐called using Guppy (version 6.3.4 + cfaa134) (Oxford Nanopore Technologies) and the PromethION sequencing data were base‐called using Dorado (version 0.3.1) (Oxford Nanopore Technologies). After base calling, duplex reads were extracted from the ONT dataset yielding a total of 1,191,939 duplex pairs. Adapters were removed using Guppy (version 6.3.4 + cfaa134, Oxford Nanopore Technologies) for MinION data and Porechop (version 0.2.4) (Wick and Volkening 2018) for PromethION data. Further methodological details are available in Appendix A (Methods). The 3 kb lambda DNA control sequence was removed using clean (v0.2.0 via nextflow 23.04.4.5881) (Lataretu et al. 2023). Since all duplex reads had a Phred quality score ≥ 7, no additional quality control filtering was applied to this dataset; however, simplex data were filtered and trimmed using Chopper (version 0.6.0) (De Coster and Rademakers 2023) specifying options ‐‐headcrop 50 and ‐‐qc 7.

After filtering, the PromethION simplex and duplex reads, as well as the MinION simplex and duplex reads, were concatenated for downstream assembly pipelines. The final ONT dataset had a mean read length of 8382 bp and mean read quality of Q18.8 (Table 1).

**TABLE 1:** Overview of filtering steps applied to Illumina and Oxford Nanopore data.

Platform	Instrument	Method	Condition	No. of reads	Bases (Gbp)	Mean length
Illumina	NovaSeq	—	Primary QC	262,988,602 pairs	79	150.0
		—	Adapter trimmed, contamination removed	233,736,388 pairs	70	149.97
Oxford Nanopore	PromethION	Simplex	Raw	9,632,490	74.69	7744.9
		Simplex	CR removed, adapter trimmed, QC filtered	1,707,688	12.24	7169.3
		Duplex	Raw	544,717	4.30	7888.1
		Duplex	CR removed, adapter trimmed	26,783	0.180	6540.4
	MinION	Simplex	Raw + adapter trimmed	7,381,142	61.43	8332.2
		Simplex	CR removed, adapter trimmed, QC filtered	1,207,050	10.46	8663.8
		Duplex	Raw + adapter trimmed	480,403	4.07	8465.0
		Duplex	CR removed	21,598	0.164	7600.0
Final ONT reads (PromethION + MinION)			Simplex & Duplex	90,090,211	76.12	8373.4

*Note:* Illumina statistics assessed via MultiQC (version 1.15) (Ewels et al. 2016); ONT statistics via NanoPlot (version 1.32.0) (De Coster and Rademakers 2023).

Abbreviation: CR, control region.

#### 2.3.2 Illumina Reads

Adapters were removed from the Illumina reads using Trimmomatic (version 0.39) (Bolger et al. 2014). Kraken2 (version kraken/2.0.7‐beta) (Wood and Salzberg 2014) was used to detect and remove sequences from archaea, bacteria, viruses and humans, using the MiniKraken2 v2 8GB database. This step resulted in 233,736,388 read pairs remaining with an average mean read quality of Q34.83 (Table 1).

### 2.4 Genome Size, Coverage and Heterozygosity Estimation

Genome size and sequencing coverage based on the filtered Illumina sequence reads were estimated using Jellyfish (v2.2.10) (Marcais and Kingsford 2012) and analysed and visualised using GenomeScope2 (Ranallo‐Benavidez et al. 2020).

### 2.5 Assemblies and Annotation

For a general illustrative overview of the genome assembly pipeline, please refer to Figure 2, below.

**FIGURE 2:**
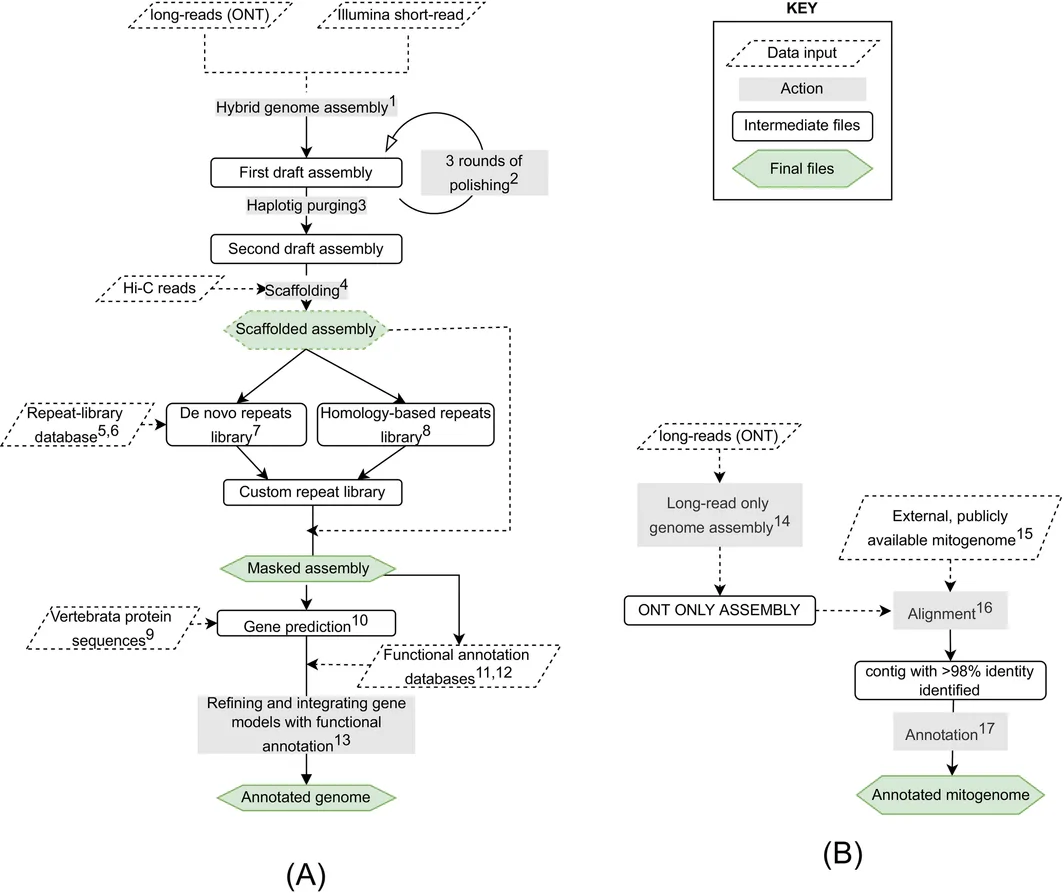
An outline of the assembly methods used to assemble the *Seriola lalandi lalandi* genome. Each step is sequentially represented; with input data in dotted outline, and resulting final data in green hexagons, illustrating the progression from raw data to the final assemblies. (A) The pipeline for developing Haku Raukura, that is, the chromosome‐level assembly for *Seriola lalandi lalandi*. The statistical results for each iteration of the genome assembly are available in Table A1. (B) The pipeline for extracting and annotating the mitogenome for *Seriola lalandi lalandi*. References for (A) ^1^MaSuRCA v3.2.9 (Zimin et al. 2013); ^2^Pilon v1.23 (Walker et al. 2014); ^3^Purge Haplotigs v1.1.2 (Roach et al. 2018); ^4^YaHS v1.1 (Zhou et al. 2023); ^5^Dfam v3.8 (Storer et al. 2021); ^6^RepBase v20181026 (Bao et al. 2015); ^7^RepeatModeler v2.0.1 (Flynn et al. 2020); ^8^RepeatMasker v4.1.1 (Smit et al. 2015); ^9^OrthoDB v11 (Kuznetsov et al. 2023); ^10^Gene prediction (Gabriel et al. 2021); ^11^SwissProt database (Apweiler et al. 2004); ^12^InterProScan 5.68–100.0 (Jones et al. 2014); ^13^AGAT Toolkit v1.4.0 (Dainat 2022); references for (B) ^14^FLYE v2.9.2‐b1795 (Kolmogorov et al. 2019); ^15^ *Seriola lalandi* isolate Seriola_lalandi_NSW mitochondrion, complete genome (GenBank accession MW309824.1); ^16^BLAST+ v2.14.1 (Camacho et al. 2009); ^17^MitoAnnotator (Zhu et al. 2023).

The Maryland Super Read Cabog Assembler, MaSuRCA (version 3.2.9) (Zimin et al. 2013) has been widely used for assembling eukaryotic genomes using hybrid short‐ and long‐read approaches, delivering consistently high‐quality results across studies (Tan et al. 2018); indeed, this method was found to be most suitable for our data. Before assembly, the Illumina sequences were not trimmed or edited as per MaSuRCA author recommendation (https://github.com/alekseyzimin/masurca). Unfiltered Illumina short‐reads and filtered ONT reads were used with recommended parameters. Methods, including the configuration file, are provided in Appendix A. The assembly generated was called the ‘First draft assembly’. The first draft assembly was subsequently put through three iterations of Pilon (version 1.23) (Walker et al. 2014) using the ‐‐frags and ‐‐unpaired options (i.e., a hybrid polishing step). The option ‐‐fix‐all was also used to ensure a comprehensive correction of assembly errors. To produce a haploid‐collapsed reference genome suitable for downstream read mapping and variant‐based analyses, PurgeHaplotigs (version 1.1.2) (Roach et al. 2018) was used to identify and remove alternative haplotigs. Parameters (−l 3 ‐m 50 ‐h 200) were selected based on the observed coverage distribution in the graphical histogram (Figure A1). Read depth for haplotig identification was estimated using Illumina short‐read mappings, as these provide more uniform and accurate coverage profiles than long‐read data for distinguishing haploid and diploid contigs.

The assembly generated is referred to as the ‘Second draft assembly’.

For Hi‐C scaffolding, the Second draft assembly was indexed and aligned to Hi‐C short reads using BWA, Burrows‐Wheeler alignment (version 0.7.17‐r1188) (Li and Durbin 2009). PCR duplicates were flagged with SAMBLASTER (version 0.1.26) (Faust and Hall 2014) using default settings, and non‐primary and unmapped aligned reads were removed using Samtools (version 1.17) (Danecek et al. 2021). Finally, YaHS, Yet another Hi‐C scaffolding tool (version YaHS‐1.1) (Zhou et al. 2023) was used to order and orientate the Second draft assembly. The minimum read mapping quality was set to 20 (−q 20) and ‐e GATC (*DpnII restriction enzyme*) was specified.

The resulting scaffolded genome is referred to as the ‘Scaffolded assembly’.

The statistics for each iteration of the assembly culminating to the Haku Raukura (final) assembly are found in Table A1.

#### 2.5.1 Contamination Screening

Contamination was evaluated using NCBI Foreign Contamination Screen (FCS) module FCS‐GX (version 0.4.1) (Astashyn et al. 2023). FCS‐GX classifies sequences as contaminants when their taxonomic assignment is different from the user provided taxonomic identifier; for this assessment, the NCBI tax ID ‐‐tax‐id 302,047 was used, which identifies *Seriola lalandi* . No contamination was identified.

### 2.6 Repeat Masking and Protein Annotation

Repetitive elements in the Haku Raukura assembly were identified using both de novo modelling and homologue‐based approaches. For de novo repeat identification, RepeatModeler (version 2.0.1) (Flynn et al. 2020) was used with the ‐LTRStruct parameter enabled. For homology‐based repeat identification, known *Actinopterygii* repeats were retrieved using the famdb.py utility of RepeatMasker (version 4.1.1) (Smit et al. 2015) from a combined database comprising Dfam (version 3.8) (Storer et al. 2021) and RepBase RepeatMasker Edition (v20181026) (Bao et al. 2015) specifying the parameters families ‐‐include‐class‐in‐name ‐ad ‐‐add‐reverse‐complement Actinopterygii. The repeat libraries generated by de novo modelling and homology‐based identification were concatenated to create a custom repeat library specific to *S. lalandi lalandi* . Genome assembly sequences were then mapped to this library using RepeatMasker (v4.1.1) (−xsmall) to classify repeat regions and produce a repeat annotation file. This process also generated a soft‐masked genome assembly. A hard‐masked version was created by replacing lowercase bases with ‘N’ characters.

### 2.7 Protein Sequence Prediction and Functional Annotation

Protein sequences were predicted using BRAKER3 (version v3.0.8 via singularity [version 3.7.3]) (Gabriel et al. 2021; Stanke et al. 2006) on the soft‐masked Haku Raukura assembly. Homology‐based gene predictions were then conducted with the OrthoDB v11 Vertebrates database (Kuznetsov et al. 2023), improving the accuracy of the predicted models through conserved domain and orthology information. Functional annotation was carried out in two sequential steps. First, protein predictions were aligned against the UniProtKB/Swiss‐Prot database (Apweiler et al. 2004) using BLAST+ (version 2.6.0) (Camacho et al. 2009). An *E*‐value cut‐off of $1\times 10^{-6}$ was used (−evalue 1e‐6), and only the top‐scoring alignment was retained by specifying ‐max_hsps 1 ‐max_target_seqs 1. Second, InterProScan (version 5.68–100.0) (Blum et al. 2021; Jones et al. 2014) was employed to identify functional domains and additional annotations, specifying the Pfam database (−appl pfam), Gene Ontology (GO) terms (−goterms) and pathway annotations (−dp).

The resulting functional annotations were integrated into gene models and refined using the AGAT toolkit (version 1.4.0) (Dainat 2022). The detailed methodology for this step is available in the Appendix A (Methods).

### 2.8 Mitogenome Sequence

The mitogenome was identified by aligning a publicly available *Seriola lalandi* mitogenome ( *Seriola lalandi* isolate Serio_lalandi_NSW mitochondrion GenBank accession number MW309824.1) to anONT‐only version of the *S. lalandi lalandi* assembly via blastn (version BLAST+/2.14.1) (Camacho et al. 2009). A contig with >98% match was found. It was annotated and visualised using MitoAnnotator (Zhu et al. 2023).

### 2.9 Synteny

To investigate genomic differentiation between the Southern Hemisphere *S. lalandi lalandi* and related populations, we performed a synteny comparison with *S. lalandi aureovittata* (RefSeq assembly GCF_021018895.1 [ASM2101889v1]). This subspecies was selected as, at the time of publication, it represented the only closely related, chromosome‐level assembly available for *S. lalandi* on NCBI; other chromosome‐level *Seriola* assemblies available (e.g., *S. dumerili* ) represented more distantly related species.

To conduct the synteny analysis, we utilised MUMMER4 (version 4.0.0.) (Marçais et al. 2018). Nucmer was used for whole‐genome alignment, specifying *S. lalandi aureovittata* (RefSeq: GCF_021018895.1) as the reference and *S. lalandi lalandi* as the query. To refine the alignment, delta‐filter was applied with a minimum identity threshold of 95% (delta‐filter ‐i 95) and minimum alignment length of 10,000 bp (delta‐filter ‐l l0000). Then, mummerplot was used to generate the visual results for the synteny alignment, using the options ‐‐filter ‐‐fat ‐‐layout. Finally, dnadiff was run on the filtered dataset to generate a report of the differences between the genomes of *S. lalandi lalandi* and *S. lalandi aureovittata*.

## 3 Results

### 3.1 Genome Size, Coverage and Heterozygosity Estimate Post Sequencing

Genome size, coverage and heterozygosity were estimated from Illumina short‐read data using *k*‐mer–based analysis. Among the *k*‐mer sizes evaluated, the 27‐mer model demonstrated the best fit, with model accuracy ranging from 92.48% to 97.01% (Table 2). Based on this model, the 588 Mbp. estimated genome haploid length was approximately 588.4–588.9 Mbp.

**TABLE 2:** *K*‐mer Analysis Statistics from GenomeScope 2.0. The model based on 27‐mers demonstrated the highest model fit, suggesting it as the most reliable for genome size estimation.

*K*	Genome length (bp)	Heterozygosity		Homozygous content		Model fit		
	Min–Max	Min (%)	Max (%)	Min (%)	Max (%)	Min (%)	Max (%)	*K*_cov_
17	281.8 M–282.6 M	5.97	17.74	82.26	94.03	83.86	91.45	96.0
21	586.8 M–587.3 M	0.48	0.49	99.51	99.52	90.75	96.62	45.7
27	588.4 M–588.9 M	0.43	0.44	99.56	99.57	92.48	97.01	42.9

Heterozygosity was calculated at 0.43%–0.44%, indicating a relatively low level of genetic variation within the genome. The heterozygous coverage of 42.9× was considered sufficient for performing genome assembly. The 27‐mer model also showed the highest proportion of homozygous content (99.56%) with minimal repeat content (32.9–32.9 Mbp), suggesting good assembly potential.

### 3.2 Final Genome Assembly

The final scaffolded assembly consisted of a total length of 644,985,034 bp in 146 contigs. Visualisation of the Hi‐C contact map confirmed 24 anchored scaffolds (Figure 3). These 24 scaffolds corresponded to 99.64% of the scaffolded assembly by base count, with an average length of 26,779.82 kb. The unanchored 122 scaffolds were shorter with an average length of 18.8 kb, covering only 0.36% of the assembly. The 24 anchored scaffolds totalled 642 Mb, with an N50 scaffold length of 34 Mbp, N50 scaffold count of 11 and a complete BUSCO (Benchmarking Universal Single‐Copy Orthologs) score of 98.6% (BUSCO [version 5.5.0]) using the actinopterygii_odb10 database (Table 3).

**FIGURE 3:**
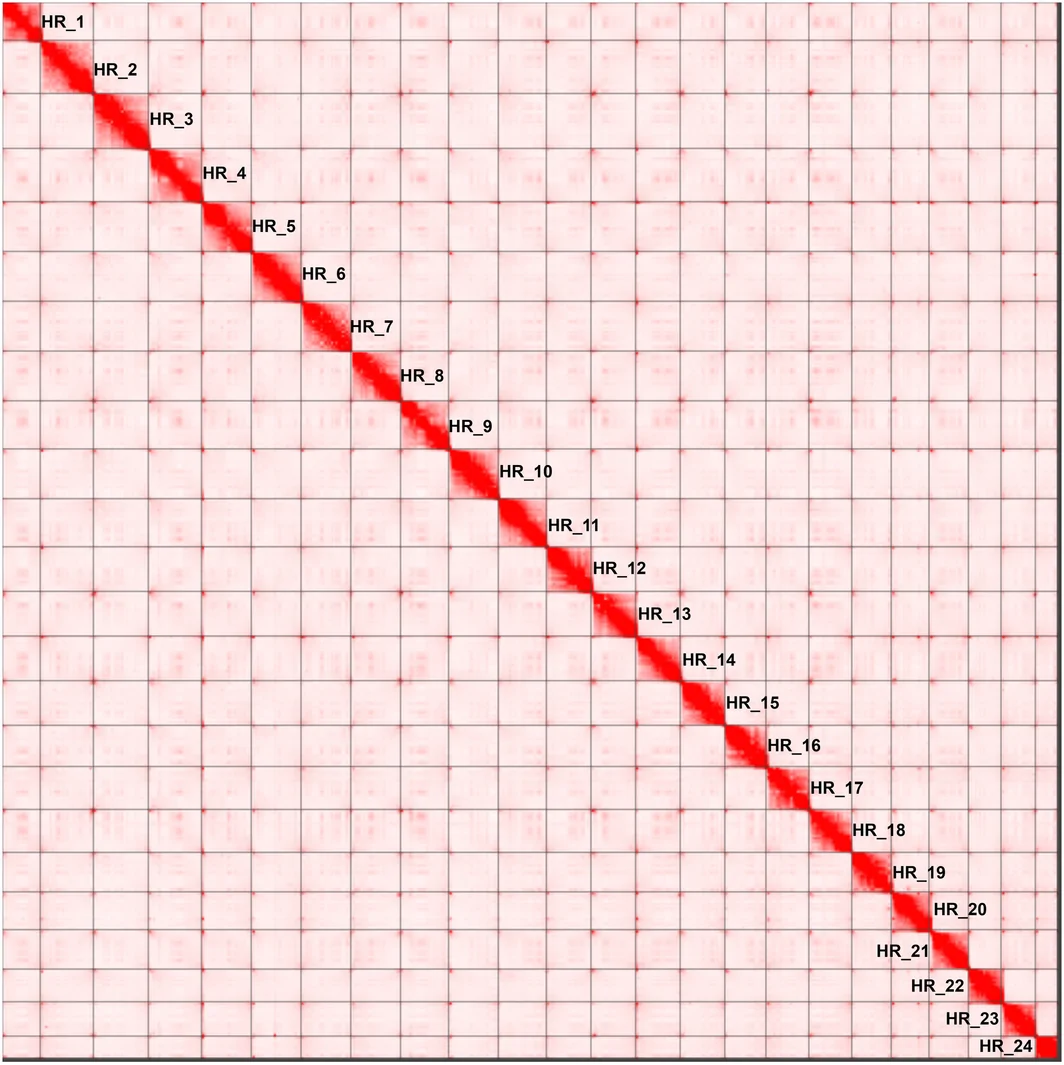
Hi‐C contact map of Haku Raukura ( *S. lalandi lalandi* ). This heatmap illustrates the interaction frequencies between different regions within the genome. Interactions are depicted by the intensity of the red colouring, with darker shades indicating higher frequencies of contact between loci. The matrix is organised such that both the *x*‐axis and *y*‐axis represent the linear progression of genomic coordinates.

**TABLE 3:** Statistics for the Haku Raukura ( *S. lalandi lalandi* ) assembly.

Metric	Value
Span (bp)	642,715,660
*N* (%)	—
GC (%)	40.81
AT (%)	59.19
Scaffold count	24
Longest scaffold (bp)	34,161,391
Scaffold N50 length (bp)	28,040,511
Scaffold N50 count	11
Scaffold N90 length (bp)	22,307,305
Scaffold N90 count	21
Complete BUSCOs (C)	98.60%
Complete and single copy BUSCOs (S)	98%
Complete and duplicated BUSCOs (D)	0.70%
Fragmented BUSCOS (F)	0.20%
Missing BUSCOS (M)	1.20%

Abbreviation: BUSCO, Benchmarking Universal Single‐Copy Orthologs.

### 3.3 Repeat Masking

The results identified 20 Mb of repeats and included a total of 16.6 Mb interspersed repeats (18.56% of the genome), 3.04 Mb satellite repeats (3%) and 1 Mb of small RNAs (0.16%). In total, 22% of the genome was masked for repeats (Table 4).

**TABLE 4:** Summary of repeat elements in the Haku Raukura ( *S. lalandi lalandi* ) assembly.

Repeat elements	Copies	Bases	Coverage of genome (%)
Interspersed repeats			
SINES	15,039	1,393,562	0.22
Penelope	80	6580	0.00
LINE	40,018	8,394,254	1.31
LTR	8,394,254	2,763,167	0.43
DNA transposons	2,763,167	2,763,167	3.92
Rolling circles	2469	814,453	0.13
Unclassified	549,317	549,317	12.55
Total interspersed repeats	11,764,344	16,684,500	18.56
Simple repeats	411,847	411,847	3.02
Satellites	1346	134,950	0.02
Low complexity	45,848	2,495,677	0.39
Total satellite repeats	459,041	3,042,474	3
Small RNA	11,972	1,056,110	0.16
Total	12,235,357	20,783,084	22

### 3.4 Protein Sequence Prediction and Functional Annotation

Protein sequence prediction identified a total of 38,487 gene models and 42,264 mRNA models for Haku Raukura. The mean gene length was 6610 bp and the mean mRNA length was 7144 bp; the mean exon length was 174 bp and the mean intron length in CDS (coding sequence) was 853 bp. Functional annotation identifies 24,600 catalogued genes. Of the predicted gene models, 24,600 (64%) were named. For the total predicted gene models, 96.1% of 3640 actinopterygii conserved genes are complete (BUSCO 5.5.0 actinopterygii_odb10 database). Similarly, 27,861 (66%) mRNA models were named, while 14,403 (34%) were unnamed. A total of 21,143 genes were assigned to at least one of the 5 queried databases (Table 5). Those remaining unnamed or ‘hypothetical’ likely represent hypothetical or poorly characterised genes.

**TABLE 5:** Annotation of Haku Raukura *S. lalandi lalandi* genes to different databases.

Type	Database	Assigned gene number
Homologue	Gene ontology	14,135
	InterPro	22,614
	MetaCyc	12,473
	Pfam	23,118
	Reactome	20,247

### 3.5 Synteny

The 24 pseudo‐chromosomes of *S. lalandi lalandi* had a clear one‐to‐one relationship to *S. lalandi aureovittata* (RefSeq: GCF_021018895.1) pseudo‐chromosomes, with 82% of bases aligning (Figure 4). A total of 16,613 one‐to‐one alignments were identified, exhibiting an average alignment length of approximately 32 kb and a high average sequence identity of 98.44%. The analysis detected 33,310 breakpoints, along with relocations (36 in the reference vs. 27 in the query), translocations (13 in both assemblies) and inversions (3 in the reference vs. 4 in the query). In total, 3 million SNPs and 5.29 million indels were observed in each assembly, highlighting both the extensive synteny and the presence of structural rearrangements between these two closely related *Seriola* genomes.

**FIGURE 4:**
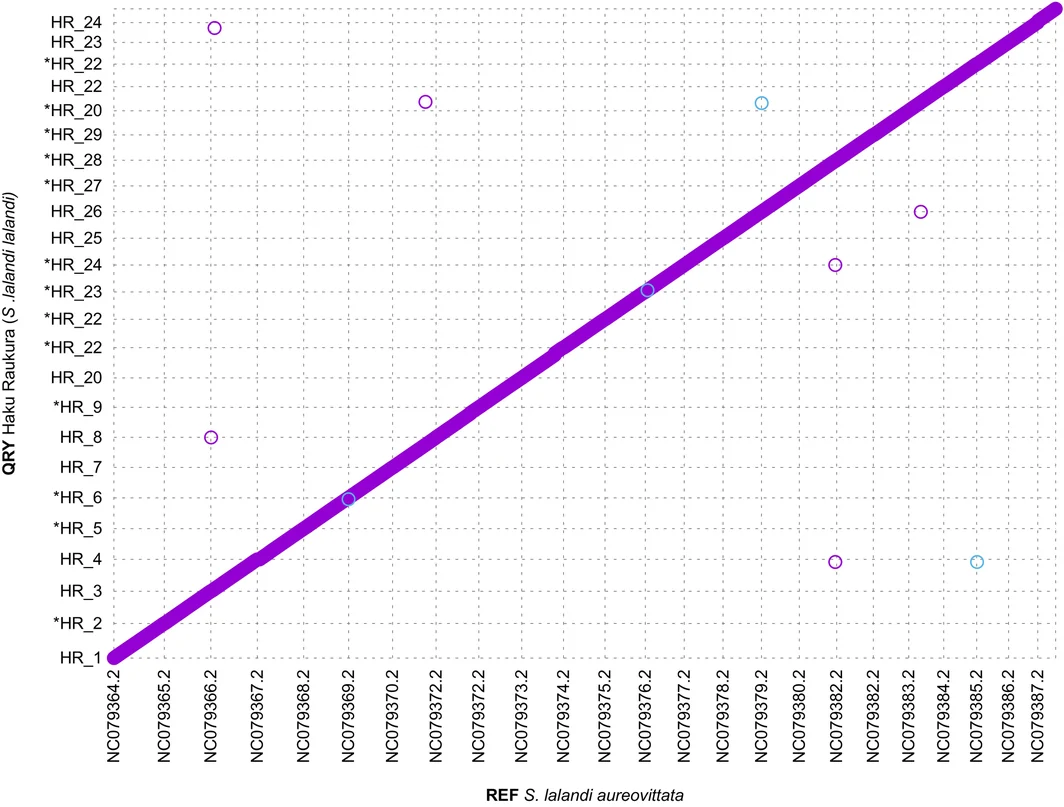
Whole genome plot of *S. lalandi aureovittata* (RefSeq: GCF_021018895.1) (reference, *x*‐axis) compared with the Haku Raukura assembly (query, *y*‐axis). Each colour dot/line indicates a match between the reference and query. Forward mappings are purple; reverse mappings are blue. Asterisked chromosomes indicate reverse‐complemented sequences (i.e., sequences that have been flipped in orientation).

### 3.6 Mitogenome Results

This contig identified was 16,535 bp in length. It was annotated and visualised using MitoAnnotator (Iwasaki et al. 2013; Sato et al. 2018; Zhu et al. 2023). There were 13 genes: 2 rRNAs and 22 tRNAs. The overall base composition was as follows: A, 26.6%; C, 30.31%; G, 17.86%; T, 25.23%. The mitogenome is shown in Figure 5.

**FIGURE 5:**
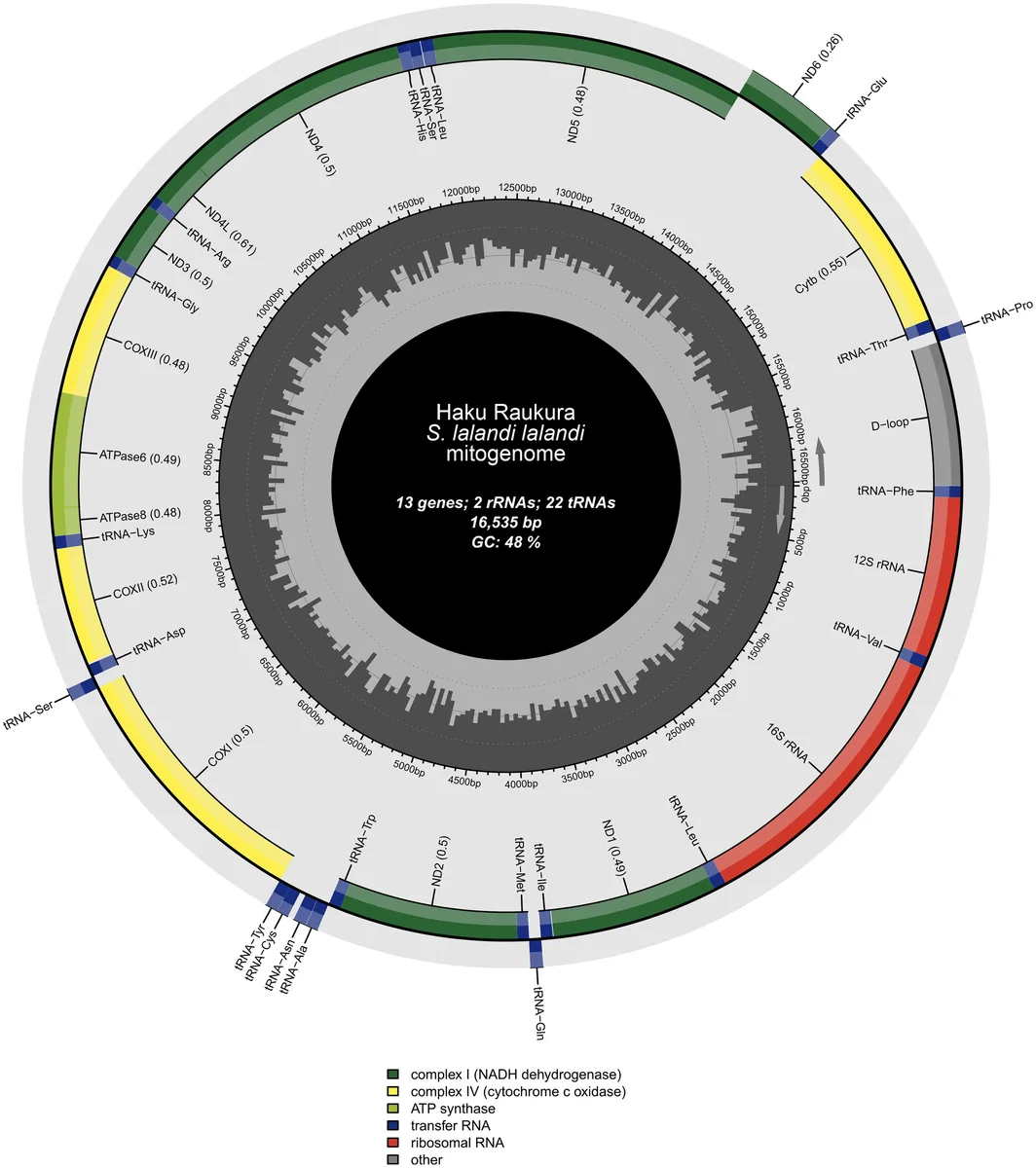
The mitogenome for Haku Raukura *S. lalandi lalandi* .

## 4 Discussion

### 4.1 Genome Quality and Relevance

In the context of other publicly available *Seriola* genomes, the final Haku Raukura ( *S. lalandi lalandi* ) assembly presented here demonstrates high contiguity and completeness, placing it among the more robust assemblies for the genus. Our scaffold N50 of 28 Mb is comparable to the other high‐quality *Seriola* assemblies released thus far (e.g., 28.5 Mb) for the Northwestern *S. lalandi aureovitata* (RefSeq: GCF_021018895.1) and substantially higher than assemblies of other *Seriola* species (e.g., *S. quinqueradiata* with 5.6 Mb). Furthermore, BUSCO analysis (actinopterygii_odb10) indicates a completeness of 98.6%, which is on par with or only marginally below the highest reported values (e.g., 99.4% for *S. lalandi aureovittata* and 99.8% for *S. dumerili* ). This indicates that the assembled pseudochromosomes for *S. lalandi lalandi* cover nearly all expected core orthologues. The total repeat content (22%) aligns with that reported for other Carangidae genomes (20%–22%), such as 20.24% for *Trachinotus ovatus* (Zhang et al. 2019) and 22.46% for *S. lalandi aureovittata* (Li et al. 2022), suggesting broadly consistent repetitive architecture across related species. Whilst the 27‐mer model estimated the genome size to 588Mbp—which is notably lower than the *C*‐value (pg) of 0.7 according to the Animal Genome Size Database (Animal Genome Size Database: Species Record id = 2705)—it is common for *k*‐mer estimated size and genome assembly size to be smaller than the size estimated with *C*‐value (Pflug et al. 2020), so this was not considered a problem. Heterozygosity was calculated at 0.43%–0.44%, indicating a relatively low level of genetic variation within the genome. This is not an uncommon range; other members of the Carangidae demonstrate comparable levels of diversity, such as 0.31% in *Trachinotus ovatus* (Zhang et al. 2019) and 0.71% in *Pseudocaranx georgianus* (Catanach et al. 2021). These results provide a baseline estimate of standing genetic variation in *S. lalandi lalandi* and reinforce the value of a population‐specific reference genome for downstream analyses. However, this heterozygosity estimate should be interpreted with caution. The sequenced individual was captive‐bred and likely represents either the first (F1) or second (F2) generation from wild‐caught broodstock. Future sequencing of wild‐caught individuals and additional captive‐derived fish would help distinguish population‐level diversity from sample‐specific variation.

Our syntenic analyses reveal that *S. lalandi lalandi* shares 98% sequence identity with *S. lalandi aureovittata*, yet exhibits structural rearrangements, numerous breakpoints and unique SNPs/indels. These findings underscore the genomic distinctiveness of the Southern Hemisphere population and highlight the importance of a local reference for capturing population‐specific variation accurately, and indeed, recent work highlights the benefits of using population‐specific reference genomes to reduce reference bias, provided assembly quality remains high (Thorburn et al. 2023). Consistent with this low heterozygosity (0.43%–0.44%) and the presence of a single dominant coverage peak, the final Haku Raukura assembly was generated as a haploid‐collapsed reference, in which alternative haplotypes were intentionally removed. This avoided artificial duplication of allelic contigs and yielded a non‐redundant genome representation. Such an approach is appropriate for the primary applications of this resource, including read mapping, variant discovery, comparative genomics and aquaculture breeding programmes—but notably, future phased or pangenome assemblies could complement this resource by explicitly representing haplotypic diversity.

### 4.2 Implications for Aquaculture

This reference genome greatly facilitates the identification of SNPs and indels in economically important genes across breeding populations of *S. lalandi lalandi* . In practice, aquaculture programmes can integrate such markers into breeding value prediction models (e.g., genomic selection), enabling earlier and more precise selection for traits that are otherwise challenging to measure (e.g., feed conversion or fillet quality). Additionally, the Haku Raukura genome has already proven useful for sex determination applications: a sex‐identification SNP in Hsd17b1 on chromosome 17 (position 15,001,654) has been pinpointed (Earth Sciences New Zealand, personal communication), allowing faster and more reliable sexing of juvenile fish.

### 4.3 Implications for Fisheries Management

The *S. lalandi lalandi* genome is also valuable for managing wild fisheries. Genomic data can complement traditional stock assessments by revealing fine‐scale population structure, genetic diversity and cryptic stock boundaries that may not be evident from morphological or tagging data. For instance, genome‐wide analyses of the related *S. dumerili* identified three genetic groups (one Mediterranean, two Atlantic), where only two had been presumed previously (Katirtzoglou et al. 2024), suggesting that genome‐wide polymorphic markers can enable novel stock delimitations. A similar high‐resolution survey across the *S. lalandi lalandi* range (e.g., New Zealand, Australia, other South Pacific populations) could identify sub‐populations requiring distinct management. By providing a template for resequencing data and marker discovery, this genome facilitates the development of conservation strategies tailored to specific genetic stocks.

## 5 Conclusion

The high‐quality *S. lalandi lalandi* reference genome presented here provides a comprehensive resource for aquaculture, fisheries management and conservation. Its high contiguity and completeness make it comparable to the best *Seriola* assemblies to date and reveal genomic features—such as unique structural variants and population‐specific SNPs/indels—that underscore the distinctiveness of Southern Hemisphere *S. lalandi lalandi* . This resolution enables more precise genomic selection strategies and breeding programmes, supports fine‐scale assessments of wild fish populations, and offers valuable insights into adaptive variation across different environments.

Future work could extend this resource by conducting detailed functional annotation of the syntenic breakpoint regions identified between *S. lalandi lalandi* and related populations, which may reveal adaptive genomic signatures underlying population‐specific divergence. As genomic research continues to expand across Carangidae species, this assembly will serve as a critical reference point for comparative studies, informing both scientific and cultural approaches to preserving the genetic health of *S. lalandi lalandi* populations.

## Author Contributions

**Carla H. Finn:** data curation (equal), formal analysis (lead), investigation (lead), methodology (lead), visualization (lead), writing – original draft (lead), writing – review and editing (lead). **Maren Wellenreuther:** funding acquisition (equal), investigation (supporting), supervision (equal), writing – review and editing (supporting). **David Chagné:** funding acquisition (equal), investigation (supporting), supervision (equal), writing – review and editing (supporting). **Tom Oosting:** data curation (supporting), writing – review and editing (supporting). **Yvan Papa:** data curation (supporting). **Alvin Setiawan:** resources (equal). **Vinko Besic:** data curation (supporting). **Peter Ritchie:** funding acquisition (equal), investigation (supporting), supervision (lead), writing – review and editing (supporting).

## Funding

This work was supported by Genomics Aotearoa, a platform funded through the Strategic Science Investment Fund of the Ministry of Business, Innovation and Employment (MBIE), Aotearoa New Zealand. The project was supported through the Indigenous Genomics Platform, with additional funding provided by Victoria University of Wellington, Wellington, Aotearoa New Zealand.

## Conflicts of Interest

The authors declare no conflicts of interest.

## Figures and Tables

**FIGURE A1:**
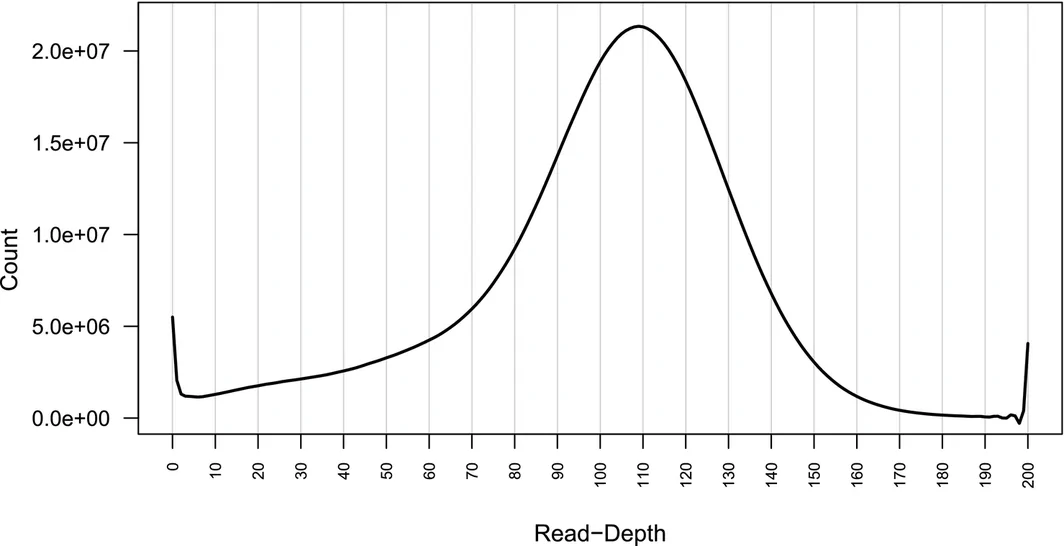
Graphical output of purge haplotigs, demonstrating 110 at the 2*n* peak. Read‐depth histogram generated by Purge Haplotigs using Illumina paired‐end reads mapped to the polished draft assembly. Illumina reads were used for coverage estimation due to their higher mapping accuracy relative to long reads. The histogram shows a single dominant peak at approximately 110×, corresponding to diploid coverage. The lack of a secondary haploid peak suggests minimal haplotig redundancy and supports the use of a haploid‐collapsed assembly.

## Data Availability

Genomic data generated in this study are available via the Aotearoa Genomic Data Repository under controlled access. Because the sampled individual originated from within the traditional territory of Patuharakeke Te Iwi Trust (Aotearoa New Zealand), access to the resulting genomic data requires approval by that authority (see Section 1.1.2). Data may be requested through: https://doi.org/10.57748/t37x‐tz81 Reviewer access is supported through applying for review‐based data access using the link above and this will be approved by the kaitiaki for the dataset (further details available at https://docs.agdr.org.nz/general_information/information_for_publishers/). The scripts used to generate these data are openly available at https://github.com/carla‐hazelf/hakuRaukura_genomeAssembly_pipeline.

## Appendix A

### Methods

#### Basecalling, Filtering and Duplex‐Calling ONT Reads

The MinION sequencing data was base‐called using Guppy (version 6.3.4 + cfaa134, Oxford Nanopore Technologies), with the dna_r10.4_e8.1 super‐accurate model. Adapter trimming was enabled during basecalling, specifying ‐c dna_r10.4_e8.1_sup.cfg ‐‐num_callers 28 ‐‐trim_adapters. After this, Duplex Tools (version 0.3.3, Oxford Nanopore Technologies) was used to identify and prepare duplex read pairs for high‐accuracy base‐calling. Duplex read pairs were extracted using pairs_from_summary, filtered with filter_pairs, and re‐basecalled using the options ‐c dna_r10.4_e8.1_sup.cfg ‐‐num_callers 28 ‐‐trim_adapters ‐‐duplex_pairing_mode from_pair_list. Raw PromethION FAST5 data was first converted to .pod5 format using the Pod5 Python tools (version 0.2.4, Oxford Nanopore Technologies). This conversion was required for compatibility with Dorado (version 0.3.1). To improve duplex base‐calling efficiency, the .pod5 files were sub‐set into per‐channel unique .pod5 files specifying ‐‐subset ‐‐columns channel as recommended by Dorado (https://github.com/nanoporetech/dorado). Duplex and simplex reads were then base‐called simultaneously using the super‐accurate model (dna_r10.4.1_e8.2_400bps_sup@v4.1.0). The resulting BAM files were validated for integrity using samtools (version 1.17) (Danecek et al. 2021), then merged, converted to FASTQ, and compressed using samtools bgzip (HTSlib version 1.17) (Bonfield et al. 2021). The dx tag in the BAM records was used to differentiate between duplex and simplex reads, defined as follows:

- dx:i:1 for duplex reads
- dx:i:0 for simplex reads without duplex offspring
- dx:i:‐1 for simplex reads with duplex offspring

In this way, samtools view (version 1.17) (Danecek et al. 2021) was used to extract the ‘dx:i:1’ tag for duplex reads. Simplex reads were extracted as both ‘dx:1:0’ and ‘dx:i:‐1’ to avoid potential loss of information. Since Dorado version 0.3.1 lacked integrated adapter removal functionality, Porechop (version 0.2.4) (Wick and Volkening 2018) was used to trim adapters from PromethION data with default settings.

#### FLYE Assembler

The following options were used FLYE (version 2.9.2‐b1795) (Kolmogorov et al. 2019): –nano‐hq –read‐error 0.03 ‐‐genome‐size 0.7 g –scaffold. The ‐‐read‐error parameter was chosen as recommended for Q20‐quality reads.

### MaSuRCA Assembler

Unfiltered Illumina sequences and filtered ONT reads were used for the assembly. This was run on MaSuRCA (version 3.2.9) (Zimin et al. 2013) with recommended parameters, automatic *k*‐mer size computation, and a config input of:PE = pe 150 22 NANOPORE = EXTEND_JUMP_READS = 0 GRAPH_KMER_SIZE = autoUSE_LINKING_MATES = 0GRID_BATCH_SIZE = 500000000 LHE_COVERAGE = 25 LIMIT_JUMP_COVERAGE = 300CA\_PARAMETERS = cgwErrorRate = 0.15 ovlRefBlockSize = 40000000CLOSE\_GAPS = 1NUM\_THREADS = 32JF_SIZE = 13000000000

Note that for the paired end parameter, the standard deviation was set to approximately 15% of the mean following MaSuRCA recommendations (https://github.com/alekseyzimin/masurca#configuration).

#### Protein Sequence Prediction and Functional Annotation

Functional annotations were integrated into gene models using the AGAT toolkit (version 1.4.0) (Dainat 2022). Specifically, agat_sp_manage_functional_annotation.pl. merged annotation results with the BRAKER3‐generated gene models, refining gene product names and descriptions. Both BLAST+ and InterProScan annotations were filtered using an *E*‐value threshold of $1\times 10^{-6}$. Predicted gene models were refined using AGAT scripts in the following order:

1. Premature stop codon removal (agat_sp_flag_premature_stop_codons.pl)
2. ORF Size filtering (agat_sp_filter_by_ORF_size.pl. ‐‐test “> = “‐‐size 50)
3. Filtering Incomplete Coding Models (agat_sp_filter_incomplete_gene_coding_models.pl)
4. Gene length filtering (agat_sp_filter_gene_by_length.pl. ‐‐test “> = “‐‐size 50)

**TABLE A1:** Assembly statistics and BUSCO completeness across major genome assembly stages, from raw draft (MaSuRCA) to final chromosome‐level scaffolds (Haku Raukura*—The longest 24 scaffolds).

Metric	First draft	Pilon 1	Pilon 2	Pilon 3	Purged haplotigs	Mapped to Hi‐C	Haku Raukura*
**Statistics**							
Span (bp)	648,299,410	648,080,790	648,016,141	647,977,682	644,971,134	644,985,034	642,715,660
*N* (%)	—	—	—	—	—	—	—
GC (%)	40.81	40.81	40.81	40.81	40.81	40.81	40.81
AT (%)	59.19	59.19	59.19	59.19	59.19	59.19	59.19
Scaffold count	390	390	390	390	273	146	24
Longest scaffold (bp)	28,950,014	28,944,476	28,943,996	28,941,487	28,941,487	34,161,391	34,161,391
Scaffold N50 length (bp)	14,193,346	14,192,664	14,192,744	14,192,743	14,192,743	28,040,511	28,040,511
Scaffold N50 count	17	17	17	17	17	11	11
Scaffold N90 length (bp)	2,230,765	2,228,891	2,228,875	2,228,872	2,272,965	22,307,305	22,307,305
Scaffold N90 count	60	60	60	60	59	2	21
**BUSCO (%)**							
Complete BUSCOs (C)	98.70	98.70	98.70	98.70	98.60	98.70	98.60
Complete and single‐copy BUSCOs (S)	97.90	97.90	97.90	97.90	97.90	98.00	98.00
Complete and duplicated BUSCOs (D)	0.80	0.80	0.80	0.80	0.70	0.70	0.70
Fragmented BUSCOs (F)	0.20	0.20	0.20	0.20	0.20	0.20	0.20
Missing BUSCOs (M)	1.10	1.10	1.10	1.10	1.20	1.10	1.20
